# Supplementary material for: Distributions of Cisco (Coregonus artedi) in the upper Great Lakes in the mid-twentieth century, when populations were in decline
Source: PLoS One. 2022 Dec 22;17(12):e0276109. doi: 10.1371/journal.pone.0276109 (PMC9778937; doi:10.1371/journal.pone.0276109)
Supplement: Attachment — Submitted filename: 02 Response to reviewers.docx [file pone.0276109.s002.docx]

**Response to Reviewer #1’s comments**

Although most of authors' responses are satisfactory, I am not yet fully convinced by the response in the letter and the corresponding revision in the text, regarding to the method to estimate catch and predict CPUE. The authors would need addition revision for this points to make clearer the method and ensure the reproducibility of the results.

On one hand, in the revised text (line 279-) and Eqn. 1, it reads that o(LNA) is the variable related to the fishing efforts, and the "first group of predictors" (in line 274-275) are o(LNA) and s_m(Mesh) in the eqn.1. On the other hand, when the authors mentioned the method to calculate CPUE (line 331-), the authors state that "gill-net Cisco CPUE as the predicted Cisco catch based on predictors that represent one unit of gill-net fishing effort, which we defined as one lift of a gang of bottom-set gill nets, with a total mesh area of 100 m2, a mesh size of 51 mm, and a fishing duration of one night". I am not sure if these two descriptions are consistent or not; how did the authors exract the necessary information (=one lift of a bottom-set gill nets with the total mesh area of 100 m2, a mesh size of 51 mm, and a one night duration) from only two variables in the model (i.e., o(LNA) and s_m(Mesh))?

Because I am not a native English speaker, I cannot judge whether these issues come from English language or (more seriously) from logical or methodological flaws. The authors may want to check again this issue to give much clearer presentation and make sure the results are reproducible.

Response: We added more explanation in the Materials and methods (starting from line 295 and starting from line 339) to clarify how CPUE was estimated. Generally, each of the Lake Superior, Lake Michigan, and Saginaw Bay full models includes different predictors for fishing effort adjustment. Thus, different inputs associated with fishing effort could be required for each selected model to estimate gill-net Cisco CPUE. For example, Lake Superior full model only included o(LNA) and sm(Mesh) as predictors for fishing effort adjustment because only data collected from gill nets set on the bottom and then lifted after one night were included in the model fitting. Thus, Catch in the Lake Superior model already represents number of Cisco caught from one lift of a gang of bottom-set gill nets, with a fishing duration of one night. Then, if the full Lake Superior model was selected, only a total gill-net mesh area of 100 m2 and a mesh size of 51 mm were required as inputs for o(LNA) and sm(Mesh), respectively, to estimate gill-net Cisco CPUE.
